# SuPAR levels in BAL fluid from patients with acute respiratory distress syndrome—a pilot study

**DOI:** 10.1186/s13054-020-03299-2

**Published:** 2020-09-25

**Authors:** Alexander C. Reisinger, Gerald Hackl, Tobias Niedrist, Martin Hoenigl, Philipp Eller, Juergen Prattes

**Affiliations:** 1 Department of Internal Medicine, Intensive Care Unit, Medical University of Graz, Auenbruggerplatz 15, 8036 Graz, Austria; 2 Clinical Institute of Medical and Chemical Laboratory Diagnostics, Medical University of Graz, Auenbruggerplatz 15, 8036 Graz, Austria; 3 Department of Internal Medicine, Section of Infectious Diseases and Tropical Medicine, Medical University of Graz, Auenbruggerplatz 15, 8036 Graz, Austria; 4 Division of Infectious Diseases and Global Public Health, Department of Medicine, University of California San Diego, 200 Dickinson Street, San Diego, CA 92103 USA

Blood levels of the soluble form of the urokinase plasminogen activator receptor (suPAR) predict mortality in patients with bacteremia and acute kidney injury [1, 2]; however, data on bronchoalveolar lavage fluid (BALF)-suPAR levels obtained from acute respiratory distress syndrome (ARDS) patients are missing.

In this study, BALF-suPAR levels were assessed in patients with ARDS (*n* = 30) and in patients without evidence of active pulmonary disease (*n* = 4). ARDS was classified according to the Berlin definition [3], independently confirmed by two intensivists, and categorized into viral and bacterial ARDS. BALF was obtained by instillation of sterile saline via fiberoptic bronchoscopy, and specimens were collected in a sterile container. suPAR levels were measured in batch using an ELISA (suPARnostic®, ViroGates A/S, Copenhagen, Denmark). The study protocol was approved by the Institutional Review Board of the Medical University of Graz, Austria (27-104 ex 14/15, amendment 2019).

Median age was similar (57 vs. 62 years, *p* = 0.49), while blood platelets (289 vs. 181 G/L, *p* = 0.04) and C-reactive protein (3 vs. 176 mg/L, *p* < 0.001) differed significantly between controls and ARDS patients. Median SOFA score was 11 points, median oxygenation index was 146 (IQR 102–186), and ICU mortality was 43% in ARDS patients. In the viral ARDS group, three patients suffered from PCR-confirmed SARS-CoV-2 infection and 11 patients suffered from severe PCR-confirmed influenza A infection, and showed no evidence for bacterial or fungal co-infection. In the bacterial ARDS cohort (*N* = 16), 10 patients had a positive bacterial culture or PCR from BALF specimens [*Legionella pneumophila* (*N* = 2), *Pseudomonas aeruginosa* (*N* = 1), *Streptococcus pneumoniae* (*N* = 2), *Stenotrophomonas maltophilia* (*N* = 1), *Chlamydia pneumoniae* (*N* = 1), *Klebsiella pneumoniae* (*N* = 1), *Escherichia coli* (*N* = 1), *Mycoplasma pneumoniae* (*N* = 1), *Brevundimonas* sp. (*N* = 1), *Staphylococcus aureus* (*N* = 1)]. In six patients, laboratory values, radiological imaging, and clinical presentation were consistent with bacterial pneumonia, but microbiological testings remained negative.

Median BALF-suPAR levels were 1.0 ng/mL (interquartile range (IQR) 0.5–1.9) in the control group and 10.8 ng/mL (IQR 5.5–26.7) in the ARDS cohort (*p* = 0.008). Median BALF-suPAR levels in patients with bacterial- and viral-caused ARDS were 16.5 (IQR 6.9–34.4, *p* = 0.003 compared to controls) and 9.5 ng/mL (IQR 1.8–15.0, *p* = 0.061 compared to controls), respectively (Fig. 1). BALF-suPAR levels were not significantly different between influenza A- and SARS-CoV-2-caused ARDS (9.1 vs. 9.8, *p* = 1.000).

**Fig. 1:**
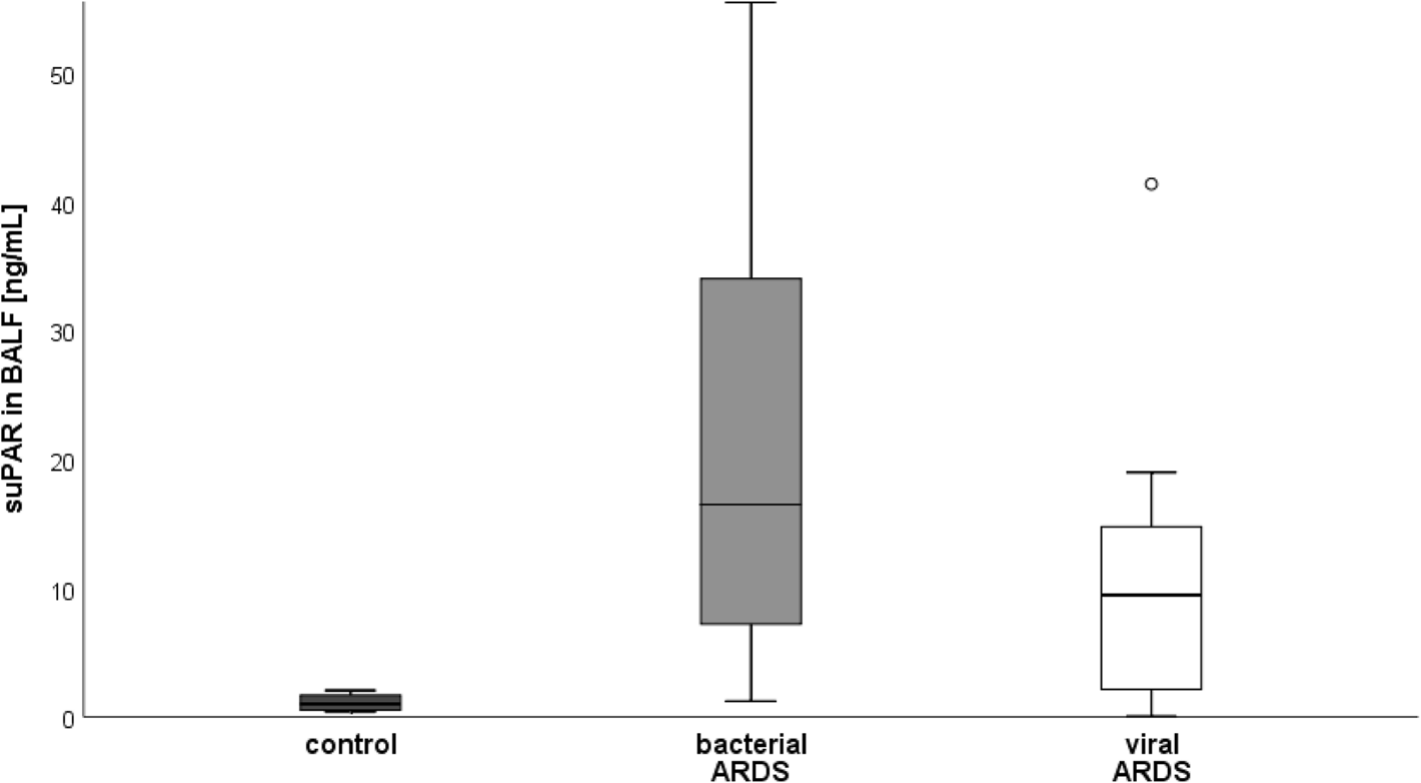
Boxplots of suPAR levels in bronchoalveolar fluid in the study population—bacterial ARDS cohort (*n* = 16; gray box plot bars), viral ARDS cohort (*n* = 14; white box plot bars), and control cohort (*n* = 4; dark-gray box plot bars). Controls received diagnostic routine bronchoscopy and bronchoalveolar lavage, and further diagnostic work-up of suspected pulmonary disease remained inconspicuous. Reasons for bronchoscopy were suspected sarcoidosis in two individuals, chronic cough in one individual, and long-term follow-up bronchoscopy after lung carcinoma resection in one individual. Statistical analyses: *p* = 0.003 between control and bacterial cohort, *p* = 0.06 between control and viral cohort, and *p* = 0.09 between bacterial and viral cohort. ARDS, acute respiratory distress syndrome; BALF, bronchoalveolar fluid; suPAR, soluble urokinase plasminogen activator receptor

BALF-suPAR levels correlated significantly with age (Spearman’s rho = 0.36, *p* = 0.05) and white blood count (*r* = 0.37, *p* = 0.04), but did not correlate significantly with SOFA score (*r* = 0.02, *p* = 0.94) or oxygenation index (*r* = − 0.05, *p* = 0.81) in ARDS patients. Furthermore, BALF-suPAR did not correlate significantly with absolute blood neutrophil count (*r* = 0.33, *p* = 0.09), blood CRP (*r* = 0.11, *p* = 0.58), or blood PCT (*r* = 0.09, *p* = 0.65).

To our knowledge, this is the first study that investigated BALF-suPAR levels in ARDS patients. We were able to show that BALF-suPAR levels are significantly higher in patients with ARDS compared to control patients without active lung disease. Patients with bacterial ARDS had a tendency to higher BALF-suPAR levels compared to patients with viral ARDS. The higher concentration of BALF-suPAR in bacterial ARDS may, at least in part, be explained by the main cellular source of suPAR, as it was recently shown that blood neutrophils are a major source of circulating suPAR [4].

One of the main limitations, which universally influences quantitative BALF biomarkers, is the wide variance in the amount of applied and retrieved fluid volume, and the volume of fluids already present within the lung. Therefore, it may be more important to use these parameters for rule-out purposes at a defined cutoff rather than rely on the exact numeric values. Additionally, in future studies, correlation of BALF and serum suPAR, as well as the influence of antimicrobial therapy or underlying diseases on suPAR levels, should be investigated.

## Data Availability

The datasets used and/or analyzed during the current study are available from the corresponding author on reasonable request.

## References

[1] Hayek SS, Leaf DE, Samman Tahhan A, Raad M, Sharma S, Waikar SS, Sever S, Camacho A, Wang X, Dande RR (2020). Soluble urokinase receptor and acute kidney injury. N Engl J Med.

[2] Raggam RB, Wagner J, Pruller F, Grisold A, Leitner E, Zollner-Schwetz I, Valentin T, Krause R, Hoenigl M (2014). Soluble urokinase plasminogen activator receptor predicts mortality in patients with systemic inflammatory response syndrome. J Intern Med.

[3] Force ADT, Ranieri VM, Rubenfeld GD, Thompson BT, Ferguson ND, Caldwell E, Fan E, Camporota L, Slutsky AS (2012). Acute respiratory distress syndrome: the Berlin Definition. JAMA.

[4] Gussen H, Hohlstein P, Bartneck M, Warzecha KT, Buendgens L, Luedde T, Trautwein C, Koch A, Tacke F (2019). Neutrophils are a main source of circulating suPAR predicting outcome in critical illness. J Intensive Care.

